# Dental caries status and related factors among 5-year-old children in Shanghai

**DOI:** 10.1186/s12903-024-04185-x

**Published:** 2024-04-16

**Authors:** Yanchen Liu, Jing Zhu, Hao Zhang, Yiwei Jiang, Huning Wang, Jin Yu, Dongxing Da, Qiwen Chen, Hongru Su, Zhengang Wu, Hongyan Shi, Jiangtao You, Xiaoli Zeng, Ying Zhang

**Affiliations:** 1 Department of Preventive Dentistry, Shanghai Stomatological Hospital and School of Stomatology, Fudan University, Shanghai, China; 2 Shanghai Key Laboratory of Craniomaxillofacial Development and Diseases, Fudan University, Shanghai, China; 3 Xuhui District Dental Centre, Shanghai, China; 4 Minhang District Dental Centre, Shanghai, China; 5 Jing’an District Dental Centre, Shanghai, China; 6 Pudong District Dental Centre, Shanghai, China; 7 Putuo District Dental Centre, Shanghai, China

**Keywords:** Early childhood caries, Risk factors, Previous dental caries experience, China

## Abstract

**Background:**

Dental caries in young children is a difficult global oral health problem. In the last decade, China has put a great deal of effort into reducing the prevalence of dental caries. This study, which is part of the China Population Chronic Disease and Nutrition Surveillance 2021, aimed to investigate the prevalence of dental caries among children aged 5 in Shanghai, China, and its associated factors.

**Methods:**

A total of 1281 children aged 5 years from 6 districts in Shanghai were selected by a stratified sampling method. The survey consisted of an oral health questionnaire and an oral health examination. The questionnaire included questions on oral health knowledge, attitudes, and behaviours. The oral health examination used WHO standards. After screening, the data were input and analysed. Chi-square tests and logistic regression analyses were used to study the relevant factors affecting dental caries.

**Results:**

The prevalence of dental caries among 1281 children was 51.0%, the dmft index score was 2.46, the Significant Caries Index (SiC) score was 6.39, and the SiC10 score was 10.35. Dental caries experience was related to the frequency of sweet drink consumption, the age of starting tooth brushing, eating habits after brushing, whether the children had received an oral examination provided by the government (*p* < 0.05), and the mother’s education level but was not related to sex, the use of fluoride toothpaste, the frequency of brushing, whether the parents assisted brushing, or the frequency of flossing (*p* > 0.05). Logistic regression analysis showed that the region of residence, eating after brushing and the age of starting brushing were associated with dental caries.

**Conclusions:**

Dental caries remained prevalent among 5-year-old children in Shanghai, China. Prevention strategies that target the associated factors including region of residence, eating after brushing, and the age of starting brushing should be considered.

## Background

Dental caries is one of the most common diseases worldwide. Globally, more than 353 million children suffer from dental caries in their primary teeth [1]. A high burden of dental caries was evident among children all over the world apparently. The World Health Organization (WHO) emphasizes the need to reduce global burden of dental caries in attaining optimal health. Consequently, in the year 2003, WHO and World Dental Federation(FDI) set global goals for oral health in 2020 to guide planners and policy makers to improve the status of oral health in their populations [2].

Dental caries occurring in children under 6 years of age are known as early childhood caries (ECC). ECC progresses at a rapid rate, and dental caries accumulate as the child grows. In addition, many studies have demonstrated that early tooth loss, malnutrition, and growth retardation due to dental caries can have a considerable impact on the quality of life of children [3, 4].In addition, children with primary dental caries are more likely to develop permanent dental caries [5, 6].

Numerous factors contribute to the development of dental caries in younger children. The interaction of environmental and genetic factors, such as the effects of oral microbiota, dietary habits, oral hygiene, salivary composition, and tooth structure, play a role in the formation and progression of caries [7]. There is a multifaceted relationship between dietary habits and dental caries, which plays an important role in the pathogenesis of dental caries. Dietary habits may be a risk factor or a preventive factor for dental caries. Lam conducted a meta-analysis and found that low parental education levels, smoking during pregnancy, nighttime breastfeeding, and frequent intake of sweetened beverages and snacks were significantly associated with the development of ECC [8]. Wong found that brushing before the age of 1 year was effective in decreasing the occurrence of dental caries, and Leroy found that parental assistance with brushing from the age of 3 years was effective in decreasing the risk of dental caries in the next 2 years [9, 10].

The prevalence of ECC varies in different regions. Developing countries have a higher dental caries prevalence than developed countries, and Asia has a higher dental caries prevalence than Europe and Latin America [11, 12]. Shanghai is an international metropolis, and its economic and cultural development is similar to that of developed countries, but the public’s concern for children’s oral health is very different from that of developed countries and regions. The fourth national oral health epidemiological survey showed that the dental caries rate of 5-year-old children in Shanghai was 68.3%, which was lower than the national average of 71.9% [3] but much higher than that in Hong Kong, Japan, and the Netherlands [13–15].

In the past two decades, China has launched various programmes to promote children’s dental health, ranging from the “Happy Mouth, Happy Family” programme organised by the National Health Commission to the comprehensive intervention programme for caries of primary teeth in preschool children [16]. However, China is a vast country with uneven development, and the oral health status of preschool children is still a serious and intractable public health problem. As an important development city in China, Shanghai has achieved rapid development in all aspects since the economic policy in the 1990s. In the past two decades, it has ranked first in domestic GDP and ranked top five in global GDP in the past 5 years. Therefore, it can represent the oral health status of the better-developed cities to a certain extent.

In the last 10 years, Shanghai has increased medical expenditure, mobilized the strength of all sectors of society, and gradually carried out oral programmes. In 2015, the Shanghai Municipal Government introduced public health measures to apply topical fluoride for dental caries prevention to reduce the rate of dental caries among children, including an oral examination once per year, topical fluoride foam applications twice a year, and long-term oral hygiene monitoring. The interventions and education have led to changes in oral health awareness and behaviour among children and parents. However, there are no studies on the prevalence of caries in Shanghai children aged 5 years in the past 3 years.

The aim of this study is to evaluate the prevalence of dental caries in primary teeth of 5-year-old school-age children in Shanghai 5 years after fluoride varnish treatment and to identify the key factors associated with dental caries. It takes a long time and costs a lot to adjust macroeconomic factors. Focusing on the role of individual factors such as children’s oral hygiene behaviors, feeding habits, and eating habits in the development of dental caries, it is of great significance to use limited resources to target prevention for children (such as brushing teeth and gargling in school). Therefore, this study also aimed to enrich baseline data and identify population-specific risk factors for this highly prevalent and preventable disease. To improve the understanding of dental caries and provide targeted recommendations for oral health care measures according to local conditions.

## Methods

### Participants

This study is part of the China Population Chronic Disease and Nutrition Surveillance 2021, which monitors the oral health status of key populations. According to the principle of cluster sampling, the Jing ‘an, Xuhui, Putuo, Hongkou, Minhang and Pudong districts were selected to conduct an oral health survey. A kindergarten-based sampling method was adopted, and 4 kindergartens in each district were selected as the investigation units, from which 50 children aged 5 were randomly selected from each kindergarten (if the number of children in a single kindergarten did not meet the sampling conditions, the number of kindergartens was appropriately expanded). The inclusion criterion for this study was healthy children aged 5. Children who were uncooperative or had a serious systemic illness were excluded.

## Survey content and methods

Two components were included: an oral health questionnaire and an oral health examination.

### Oral health questionnaire

Each questionnaire was collected by face-to-face interviews with parents of children at the site of the oral health examination. The questionnaire mainly included oral health knowledge, attitudes and behaviours, oral disease experience, and oral health service utilization. The informed consent form was signed by the child’s guardian and kept centrally by the surveyor.

### Oral health examination

Caries, missing teeth and fillings were recorded. Dental caries were determined according to the criteria recommended by the WHO [17]. The examination was carried out visually under artificial light, combined with a 0.5-mm ball-tipped CPI probe and a disposable dental mirror. Instruments included a plane mouth mirror and CPI probe, with the aid of a cotton swab to remove food if necessary. A tooth was recorded as decayed (dt) when a lesion had an unmistakable cavity, undermined enamel, or a detectably softened floor or wall; a tooth that was sealed but also decayed, was included as well. A tooth was recorded as missing(mt) when it was extracted due to caries. For missing primary teeth, this score should be used only if the subject is at an age when normal exfoliation would not be a sufficient explanation for absence. A tooth was recorded as filled (ft) when it was permanently filled without caries. Only the crown of the child’s teeth was examined, not the roots.

### Indices used

In this study, the following indicators were used: the prevalence of dental caries in the primary teeth, number of dental caries, extractions or fillings due to dental caries (dmft), Significant Caries Index (SiC) score, calculated as the mean of the top 1/3 of dmft scores, and SiC10 (mean of the top 10% of dmft scores) [18, 19].

### Quality control

A total of 6 examiners who were qualified as dental practitioners and had been practising dentistry for at least 3 years received uniform training and underwent standard consistency testing. During the oral health examination, survey respondents were examined by another examiner according to a 5% review rate. In the on-site survey, before each respondent left the site, the enumerator conducted a thorough check of all the contents of the questionnaire. If errors and omissions were found, information was added and corrected. After carefully verifying that there were no errors before signing for acceptance, the report was sealed. Kappa values were calculated and inter- and intra-examiner reproducibilities were > 0.80.

### Analysis methods

Two independent surveyors entered the data by using EpiData (EpiData Association, Odense, Denmark), and the two sets of data were compared. If inconsistencies were found, the original data were rechecked and amended accordingly. Then, all statistical analyses were performed using SPSS Statistics for Windows (version 27.0, IBM Corp. Armonk, NY, USA). The frequencies of the variables were calculated by descriptive statistical analysis. The chi-square test was used to assess the association between dental caries prevalence and the independent variables (sex, region, frequency of sweets consumption, frequency of brushing, frequency of flossing, frequency of toothpaste use, level of parental education and having received government examinations). *P* < 0.05 was considered statistically significant. Logistic regression analysis was used to examine all potential variables associated with dental caries experience.

## Results

Of the 1281 respondents (Table 1), 648 (50.6%) were boys and 633 (49.4%) were girls, with a mean age of 5.5 ± 0.3 years. A total of 653 had dental caries, with a dental caries rate of 51.0%. Among them, 342 males had dental caries, with a caries rate of 52.8%, and 311 females had dental caries, with a dental caries rate of 49.1%.

**Table 1:**
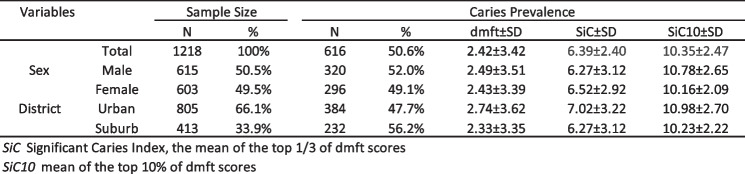
Descriptive characteristics of the sample of preschool children

**Fig. 1:**
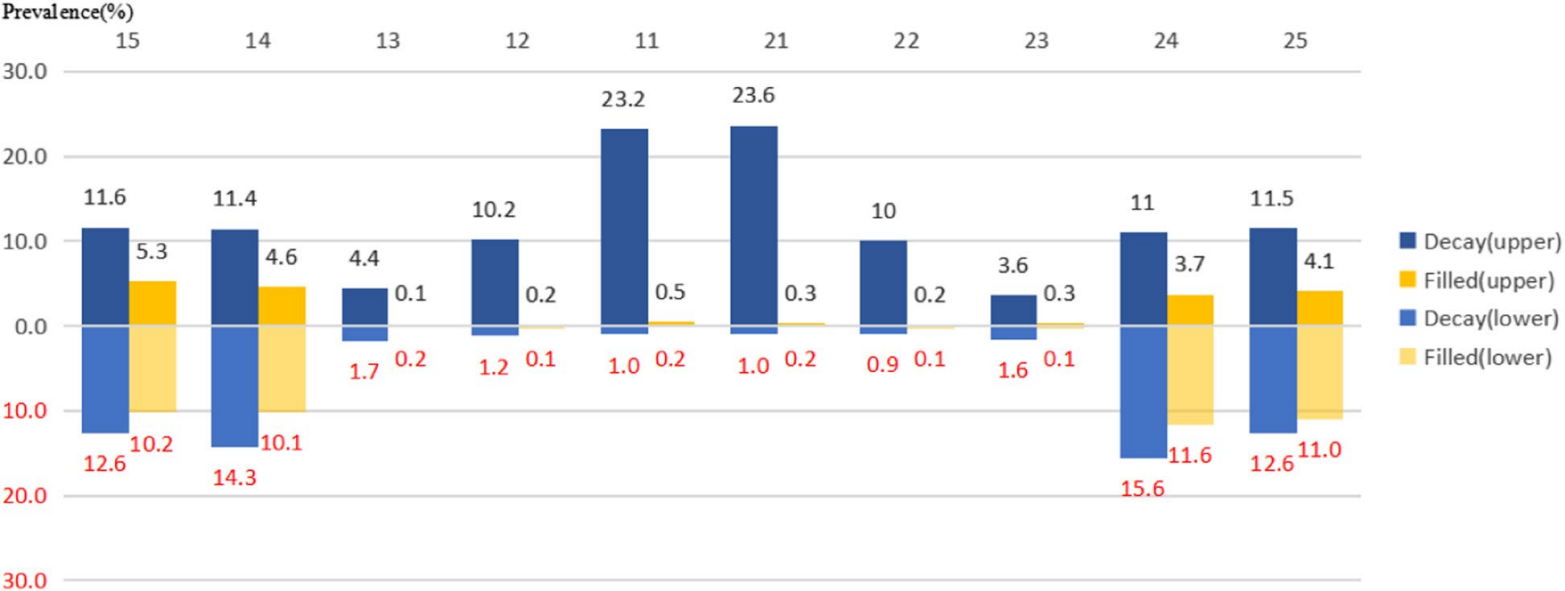
Distribution of teeth with df dental caries by tooth position

Figure 1 shows the proportion of decayed and filled teeth at each tooth position. The X-axis is the position of the primary teeth in the upper and lower dental arches. The Y-axis is the percentage of decayed (blue) and filled (yellow) teeth at each tooth position. The maxillary central incisors had the highest dental caries prevalence (23.6%), while the mandibular bilateral primary molars had the highest dental caries filling rate (11.6%).

Table 2 presents the correlation between dental caries and oral health-related behaviours in children. There was no correlation between having dental caries and eating desserts (including cakes, biscuits, chocolate, etc.), but there was a correlation with consuming beverages (such as milk, yogurt, milk tea, soya milk, etc.) (*p* < 0.05). There was a significant correlation between dental caries and the age of starting teeth brushing and whether the children ate after brushing (*p* < 0.001) and a correlation of the mother’s education level with whether the children had undergone government-provided oral examinations (*p* < 0.05).

**Table 2:**
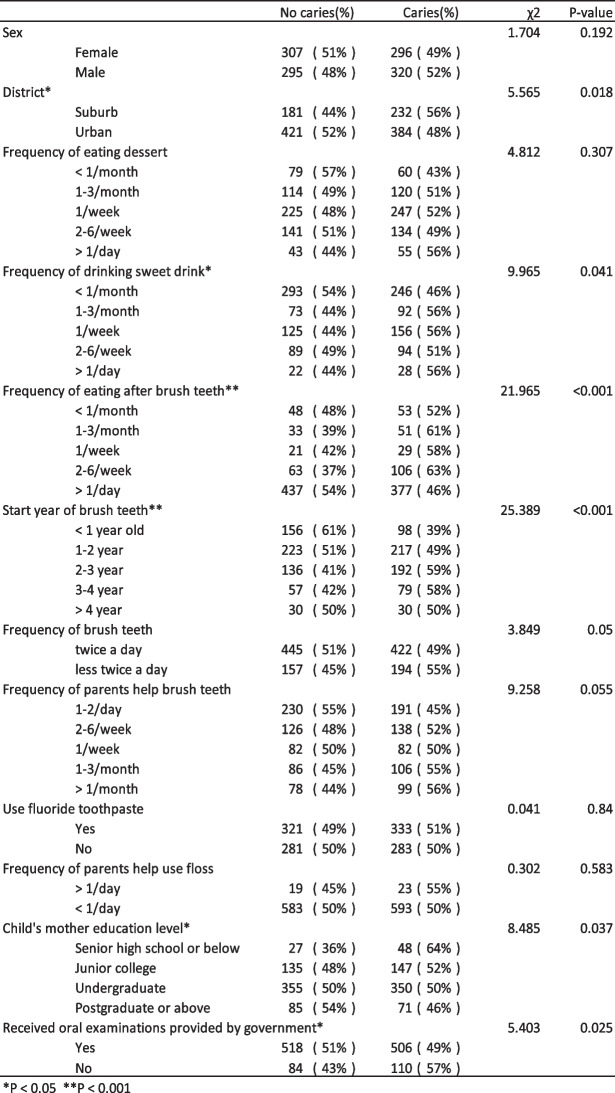
Chi-square test for the relationship between the prevalence of dental caries and selected variables

Logistic regression analysis of the variables with *p* < 0.05 revealed (Table 3) that the district, eating after brushing and age at starting teeth brushing were significant risk factors associated with dental caries. Children living in suburban areas were more likely to have dental caries than children living in urban areas (odds ratio 1.339, 95% confidence interval: 1.04–1.71). The risk of dental caries was also higher for those who ate after brushing every day than for those who never ate after brushing at night (odds ratio 1.83, 95% confidence interval: 1.29–2.59).

**Table 3:**
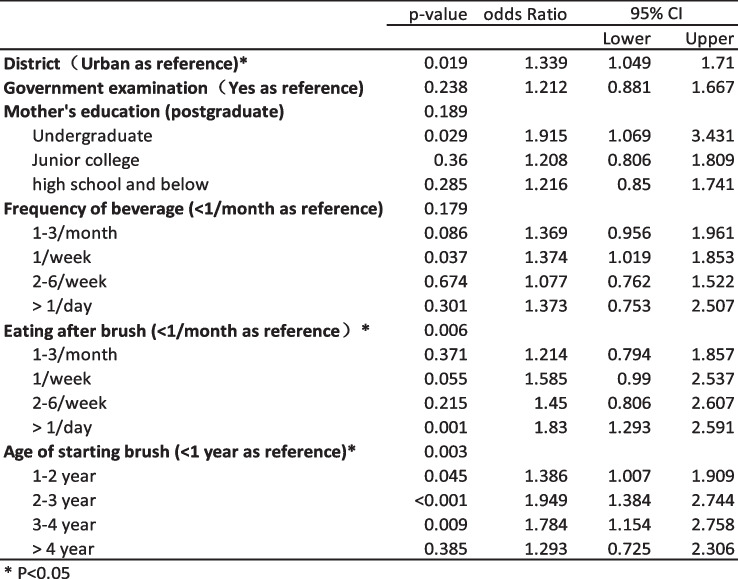
Logistic regression analysis

## Discussion

Compared with the fourth national oral health epidemiological survey, the dental caries rate of 5-year-old children in Shanghai decreased from 68.3 to 51.3%, and the dmft index score also decreased from 3.12 to 2.46, which may be closely related to the fluoride application programme in the past 5 years; furthermore, the examination has helped to increase parents’ attention to children’s oral health. However, the results of this survey are still higher than those of developed countries such as Sweden and Singapore [20, 21]. According to a study, the highest prevalence of ECC is found in the 3- to 4-year-old age group [22]. This suggests the need to advance the age of dental caries prevention to 3 years of age or even at birth.

The SIC score of 5 years old in this survey is 6.39, which is higher than that of Germany, Greece and India [2, 23, 24], and lower than that of Spain and Turkey [25, 26]. Compared with dmft, SIC can better reflect the urgency of dental treatment for children with multiple dental caries. The sic calculated as the mean of the top 1/3 of dmft scores, the higher the number, the more carious teeth the children have. In the future, more efforts should be devoted to the identification of high-risk groups of dental caries, early prevention and early treatment.

The correlation among teeth brushing frequency, parent-assisted teeth brushing and dental caries has been confirmed in several articles [27–29], and no significant difference in this paper can be summarized as substandard brushing methods or poor parent-assisted teeth brushing; many parents stated that their child’s brushing alone tends to be completed in less than a minute, that parent-assisted brushing is often difficult for children to cooperate with and that the posterior teeth are difficult to reach, which tends to cause a gagging reaction. This is consistent with the findings in the study by Boustedt et al. [30] .It is necessary to refine the length of brushing time, brushing method, etc., in future studies to help compare the results between different studies.

There are several possible explanations for why fluoride toothpaste is not associated with dental caries. First, according to the survey, only half of the children were able to ensure that they brushed their teeth twice a day and that their parents assisted in brushing their teeth. Brushing is mainly a mechanical friction to remove plaque from the teeth, and if the brushing is not effective, the use of toothpaste is not effective in preventing dental caries. This is the same as in the study by Boustedt et al. [30]. Second, the fluoride content of toothpaste used in each family is very different; the fluoride content of toothpaste for children in China is 500 ppm, and a Cochrane review of fluoride toothpastes mentions that only toothpastes greater than 1000 ppm have a significant effect on the prevention of dental caries [31]. Finally, the amount of fluoride toothpaste used varies widely, with the AAPD recommending the use of pea-sized toothpaste for children under six [32], but in practice, many parents use far less than needed. All of the above results have an impact on the statistics.

In this study, we found that the dental caries rate of children in the urban area of Shanghai was lower than that in the suburbs, which is consistent with the findings of Du et al. [3], and the possible reasons are related to the lower economic level of the suburbs compared with urban areas, the unbalanced distribution of health care resources, and the inconvenience of transport [27].

Parents’ education level was correlated with dental caries prevalence, which is consistent with the findings of Lam et al. [8]. This is because the higher the parents’ education level, the more knowledge they may have about oral health, the more they care about oral health, and the fewer dental caries their preschool children will have. Parents in Shanghai generally have higher education levels, and only 6.3% of mothers had an education level of high school and below, so there was no significant difference in the logistic regression results.

There was a statistically significant difference in the habit of eating after brushing, which is consistent with the national survey [3]. Eating after brushing is equal to not brushing before bedtime. Thererecognised are a number of factors that influence the development of dental caries: (1) cariogenic microorganisms, (2) fermentable carbohydrates (substrate), (3) susceptible tooth surfacessurface/host and (4) time. Not brushing before bedtime means that the food on the teeth surface continues to provide nutrients for the cariogenic microorganisms in the mouth, and in addition, due to the low degree of mineralization of the primary teeth, the intake of viscous and highly sugary foods accelerates the progression of dental caries.

The age of starting teeth brushing was significantly correlated with dental caries, and the later the age at which brushing began, the higher the incidence of dental caries. Nishimura found that brushing at 18 months of age effectively reduces the likelihood of dental caries over the next 2 years [33], Wong et al. also concluded that brushing should begin no later than 1 year of age [9], and Boustedt et al. concluded that the oral biofilm should be exposed to fluoride ions more often than once per day to maximize its dental caries-preventive effect.

The frequency of consumption of sweet drinks and sweets has been shown to be significantly associated with dental caries in previous studies, but only sweet drinks showed a statistically significant difference in the chi-square test in the current survey. The reason for this may be that parents in Shanghai are generally highly educated and have dental awareness and are less likely to allow their children to consume sweets, with only 7.8% consuming desserts more than once a day and 3.9% consuming sweet drinks more than once a day.

The present investigation found government examinations were no significant in the logistic regression analysis after controlling for confounding factors. The meaning of government examinations is to check after obtaining the consent of the guardian, and to give the feedback of dental caries to the parents at the end of the examination. This process can help parents recognize the importance of oral hygiene and assist their children in developing good oral habits.

There are still some limitations in this study. First, although the frequency of tooth brushing and the use of fluoride toothpaste were studied in the survey, the duration and method of tooth brushing could be more specifically detailed to better analyse the association with dental caries. Second, this was a cross-sectional survey, which could only determine the oral health status of children in Shanghai in 2021, and further analysis of the effects of oral health measures necessitates still requires prospective studies.

## Conclusion

Dental caries remained prevalent among 5-year-old children in Shanghai, China. Prevention strategies that target the associated factors including region of residence, eating after brushing, and the age of starting brushing should be considered.

## Data Availability

The datasets analysed in the current study are available from the corresponding author upon reasonable request.

## Abbreviations

SIC: Significant Caries Index
SIC10: Mean of the top 10% of dmft scores
ECC: Early childhood caries
dmft: Number of dental caries, extractions or fillings in primary teeth due to dental caries
